# Interpersonal violence in a deprived Scottish urban area with aggregations of physical health risks and psychiatric morbidity: an ecological study

**DOI:** 10.1186/s12889-021-11167-z

**Published:** 2021-06-12

**Authors:** Jeremy Coid, Yingzhe Zhang, Simone Ullrich, Jane Wood, Vishal Bhavsar, Paul Bebbington, Kamaldeep Bhui

**Affiliations:** 1 Mental Health Center and Psychiatric Laboratory, the State Key Laboratory of Biotherapy, West China Hospital of Sichuan University, Chengdu, Sichuan China; 2 Wolfson Institute of Preventive Medicine, Queen Mary University of London, London, UK; 3 Harvard Chan School of Public Health, Harvard University, Cambridge, USA; 4 School of Psychology, University of Kent, Canterbury, UK; 5 Department of Psychosis Studies, Institute of Psychiatry, Psychology and Neuroscience, King’s College London, London, UK; 6 Department of Mental Health Sciences, University College London, London, UK; 7 Department of Psychiatry & Nuffield Department of Primary Care Health Sciences Medical Sciences Division, University of Oxford, Oxford, UK

## Abstract

**Background:**

Glasgow, Scotland, has previously shown exceptional levels of violence among young men, shows aggregations of health conditions, with shortened life expectancy. Health conditions can be both causes and consequences of violence, of shared community-level socio-economic risk factors, and can result from large-scale social forces beyond the control of populations with high levels of violence. The aim of the study was to provide an in depth understanding of the Public Health problem of violence among young adult men in Glasgow East.

**Method:**

Ecological investigation of violence and its associations with health conditions in areas of contrasting socioeconomic deprivation. National survey of 1916 British men aged 18–34 years, augmented by a sub-sample of 765 men in Glasgow East (GE). Participants completed questionnaires covering current physical and sexual health, psychiatric symptoms, substance misuse, lifestyle, and crime and violence.

**Results:**

The 5-year prevalence of violence was similar in both surveys but fights involving weapons (AOR 3.32, 95% CI 2.29–4.79), gang fights (AOR 2.30, 95% CI 1.77–2.98), and instrumental violence supporting criminal lifestyles were more common in GE, where 1 in 9 men had been in prison. Violent men in both samples reported poorer physical and sexual health and all types of psychiatric morbidity except depression, with multiple high-risk behaviours for both future poor health and violence. Associations between drug and alcohol dependence and violence in GE could not be entirely explained by deprivation.

**Conclusion:**

Violence in deprived urban areas is one among many high-risk behaviours and lifestyle factors leading to, as well as resulting from, aggregations of both psychiatric and physical health conditions. Poverty partly explained raised levels of violence in GE. Other factors such as drug and alcohol misuse and macho attitudes to violence, highly prevalent among men in this socially excluded community, also contributed. Multi-component preventive interventions may be needed in deprived areas and require future investigations into how multiple co-existing risk factors produce multimorbidity, including psychiatric disorders, substance misuse, poor physical health and violence.

**Supplementary Information:**

The online version contains supplementary material available at 10.1186/s12889-021-11167-z.

## Background

Violence has fallen in the UK and many other countries [1]. However, within countries, violence is unevenly distributed between locations and socio-economic sub-groups. Although the overall rate of recorded violence has fallen, for some UK communities the public health problem of violence has by no means subsided. Violent conviction rates in teenage males have declined in Scotland, but older men still show high levels of violence [2]. From the nineteenth century, Glasgow has had a history of violence associated with gangs, exacerbated by religious sectarianism [3]. Following industrial decline in the 1960’s, Glasgow had more street gangs than other UK cities. By the 1980s, Scotland, particularly Glasgow, had the highest homicide rate in western Europe [4], although this has since fallen. The overall annual rate of assaults in Scotland, whilst considerably larger, shows close correlation when observed over time to homicide rates and to fluctuations in those rates. Homicide rates among men in Scotland fell from 1974 until the mid-1980s, then rose until 1991. This was followed by a dramatic increase and homicide remained at a high rate until 2006, after which rates steadily fell. In contrast, rates among women had slowly fallen across this entire period and women in Scotland did not contribute to the rises and falls in homicides observed in Scotland. Rates were highest among men 20–24 years and deaths were mainly due to stabbings. These fluctuations observed in homicide rates among men took place almost entirely within the most socio-economically deprived areas of Scotland which show their highest concentration in Glasgow. The observed increase in homicides up until 2006 were paralleled by an increase in deaths related to alcohol and drugs which have not shown a similar decline and have continued to increase [4, 5].

Violence is usually considered a criminal justice rather than a health problem. However, it cannot be viewed in isolation from major social changes adversely affecting physical and mental health, especially in Scotland. Health problems and life expectancy had also deteriorated in the most deprived areas by the 1980s [6]. Life expectancy trends have stalled across the UK since 2012 and Scotland has the shortest life expectancy in Western Europe driven by high rates of cancer, suicide, alcohol-related causes, and drugs-related poisonings, with no signs of improvement [6]. Socio-economic deprivation is exceptionally high in parts of Glasgow and some other Scottish cities, but not all of Scotland’s health problems or mortality can be explained by deprivation [7]. The “Scottish effect” persists after taking into account socioeconomic deprivation and health behaviours such as smoking, physical activity, and diet. Drug-related deaths in Scotland are the highest in Europe, particularly in Glasgow [8], and have now exceeded the USA, with residents three times more likely to receive inpatient care for psychiatric disorders, mainly for drug-related conditions [9]. This aggregation of physical and mental health conditions in parts of Scotland may well be due to lifestyles involving multiple, adverse, health-related behaviours which include violence. Indeed, violence could be a key marker within this wider network of morbidity. However, it is important to understand whether violence, in the context of an aggregation of associated health conditions, is explained primarily by area-level effects of severe socioeconomic deprivation or whether other factors at the individual, interpersonal and community level, specific to impoverished areas of Scotland, explain this aggregation.

## The public Health problem of violence and the ecological model

The World Health Organization defines violence as ‘The intentional use of physical force or power, threatened or actual, against oneself, another person, or against a group or community, that either results in or has a high likelihood of resulting in injury, death, psychological harm, maldevelopment or deprivation [10] and where the central concept is the intention to cause harm [11]. In this study, we focus on interpersonal violence between young adult men because this is the most prevalent form of violence internationally, the largest global public health problem, and results in the largest burden of care [12]. The public health approach to violence has become increasingly predominant in global responses to violence [13], and has been strongly promoted by the World Health Organization [14]. This approach to violence is described as collectivist in approach, contrasts from a standard health approach, and aims for prevention and minimization of harm [15]. Considerable emphasis is placed on the situation violent persons are in which make it difficult to make healthy choices and avoid unhealthy exposures and where there are considerable overlaps between violence and victimization. For example, the downward spiral of childhood exposures to maltreatment, poor parenting, and domestic abuse, early oppositional disobedience, school truancy, early drug and alcohol use and escalating involvement in violence, which are often seen in individual case studies of violence, also need to be considered in the context of structural violence [16] imposed on the communities from which these young people come [15]. Large scale political, economic, and cultural forces give rise to clustered epidemics of various diseases which can occur in situations of changing political and economic conditions, shifting ecological and environmental conditions, and altering demographics and social behaviours [17]. This implies that when studying violence in the context of Glasgow, it is necessary not only to study the effects of individual and neighbourhood factors that impact on aggregations of health conditions within this area of concentrated deprivation, but also to consider the large-scale social forces beyond the control of the populations that resulted in these aggregations.

**Fig. 1:**
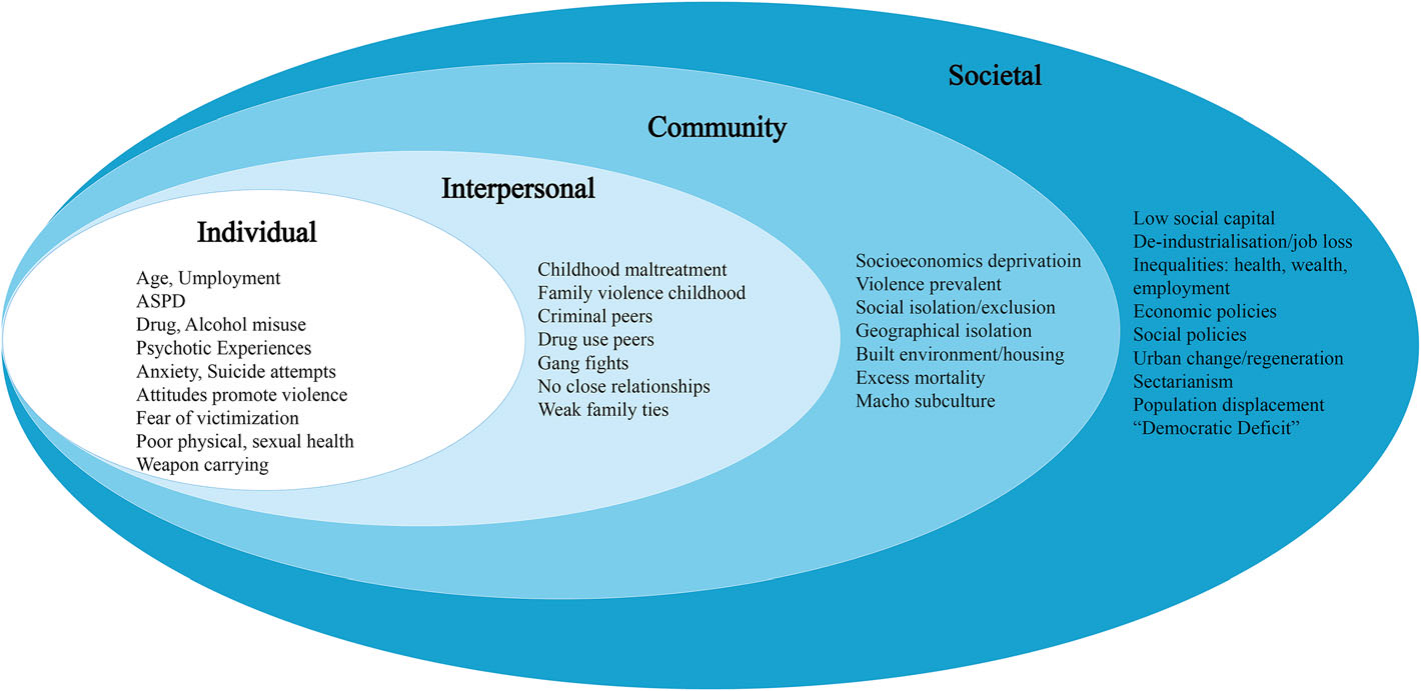
Ecological Model of Violence among Young Adult Men in Glasgow East

The public health approach describes a broad set of ways of understanding and intervening on violence and its health impact which focuses on the causes of incidence rates/trends in whole populations, rather than focusing narrowly on the causes of individual cases [18]. To do this, the WHO has recommended an ecological model of assessing violence at more than one level [19]. Socio-ecological models were developed to understand dynamic interrelationships between personal and environmental factors. Originally used as a framework for studying human development, it was argued that the entire ecological system in which growth occurs should be taken into account [20]. Prevention of violence requires understanding of the factors which promote violence and the socio-ecological model is intended to consider the complex interplay between individual, relationship, community and societal factors that put people at risk of both experiencing and perpetrating violence [19]. The overlapping rings of the model are intended to show how factors at one level influence those at another level (see Fig. 1). The first level identifies biological and personal history factors that increase the likelihood of becoming a victim or perpetrator of violence. The second level examines close relationships that may increase the risk of experiencing violence as a victim or perpetrator. The third explores the settings, such as schools, workplaces, and neighbourhoods in which social relationships occur and seeks to identify the characteristics of these settings that are associated with becoming victims or perpetrators of violence. The fourth level examines the broad societal factors that help create a climate in which violence is encouraged or inhibited and can include large-scale social forces that lead to aggregations of health conditions together with violence in certain locations. These factors include social and cultural norms that support violence as an acceptable way to resolve conflicts. Other large-scale societal factors include the health, economic, educational, and social policies that help to maintain economic or social inequalities between groups in society. An important use of this multi-level framework is to identify and cluster intervention strategies based on the ecological level in which they act.

A major aim of ecological studies is to generate or test etiological hypotheses, but it can also be a method to identify factors associated with violence which are suitable for targeted and/or population prevention strategies [21]. We used an Ecological model to investigate the multiple associations between violence and environmental context in a socially excluded sub-group of Scottish men. The Ecological model of violence explores the relationship between individual and contextual factors and considers violence as the product of multiple levels of influence on behaviour [19]. Whilst some risk factors may be unique to a particular type of violence, the various types of violence more commonly share a number of risk factors. Violent men may consequently report multiple forms of violent behaviour and victim types, for example violence against strangers as well as increased risk of intimate partner violence. This method is used in Public Health rather than psychiatric investigations of violence, where it is not expected that simple, direct relationships will be consistently found that can be tested between single categories of psychiatric morbidity, physical ill-health and sub-types of violence. Each type may be influenced by multiple risk factors. Furthermore, associations at the individual level may be bi-directional. Some may be specific for violence, others specific for psychiatric or physical health morbidity. However, all may be the outcome of the same or similar risk factors operating at a different level in the ecological model.

The aims of this paper were to conduct an ecological investigation of (i) the prevalence of violence, attitudes towards violence, and associated criminality among young Scottish men residing in a socioeconomically deprived urban area compared to men in the general population of Britain; (ii) associations with adverse childhood experiences, violence-related peer influences, and lifestyle factors; (iii) associations with physical health, sexual health, substance misuse, and psychiatric morbidity. For each of these domains, we also aimed to investigate whether our findings were accounted for by socioeconomic deprivation and unemployment.

## Method

### Study participants and sampling

We used data from the Second Men’s Modern Lifestyles Survey carried out in 2011 using random location sampling, an advanced form of quota sampling shown to reduce biases introduced when interviewers are able to choose locations to sample from (see supplementary file). A National survey derived a representative sample of young men aged 18–34 years from England, Scotland and Wales. We compared a boost survey of men of the same age range from GE for comparison purposes because of exceptionally high levels of recorded health, social problems, and reduced life expectancy in that location and to test the hypothesis that higher levels of violence and specific forms of violence are more likely to be found in this location. Identical sampling principles were used for both surveys. This age range and a male sample were originally chosen to investigate risk factors for serious violence, substance misuse, and psychopathology at the population level as one component within a programme of research to improve risk management of violence [22]. Additional boost samples included hard-to-reach participants, including those from areas with exceptional characteristics and known to have high levels of violence, which included GE.

The statistical reliability of random allocation sampling depends both on strictly defining the selection of the sampling points as well as in setting representative quotas at each point, then meeting these quotas. Within each Government Office Region, all output areas (OA) (averaging 150 households, and about which all demographic profiling information is known) were selected and listed in descending order of ACORN (A Classification Of Residential Neighbourhoods) [23] type to place the most affluent OAs at the top of the list and the least affluent at the bottom. This applies a purely random variable into the selection of sampling locations. The total number of eligible male adults in each OA were then cumulated down the list. Using a random start and fixed sampling interval, the required number of OAs were selected. This process produces a sample of OAs with a probability of selection proportionate to size and was designed to produce a representative sample by ACORN type. After the total number of OAs are selected, interviewers are required to achieve a set number of interviews with eligible targets at each. All addresses that lay within selected OAs were potentially available for interview. With OA information cross-referenced against full address lists, interviewers were supplied with every single address that was eligible within each OA. A quota sheet was provided for each selected OA, which reflected the actual composition of eligible residents according to standard demographic criteria. These would include socio-demographic characteristics such as gender, ethnicity and working status (in addition to age). Interviewers were required to interview a sample profile that matched exactly that of the eligible OA population profile using the then up-to-date Office of National Statistics (ONS) population estimates information. This ensured that the sample was demographically representative at the micro-level, as well as geographically representative of males in the general population. If a participant refused to complete the questionnaire (approximately 23% of all participants approached in similar studies), or was absent, another was located in the area with exactly the same demographic profile (age and social class) until the quota was filled. Compliance with this procedure produced a fully representative data set for both the national survey and from GE. Informed consent was obtained from respondents. Pencil and paper self-report questionnaires were administered at home, with the respondent left to complete the questionnaire in their own time. The researcher either returned later that day or the next. Each questionnaire took approximately 45 min to complete. Participants were given £5 on completion of the questionnaire.

The sample included 2656 men, aged 18–34 years; 1897 (71.5%) were from the representative survey, and 759 (28.5%) were from GE. Identical sampling principles were used for both surveys (see supplementary file).

### Survey measures

#### Violence, violent attitudes, and child maltreatment

All participants were questioned about violent behavior using questions from previous UK surveys [24].

Characteristics of Violence: They were asked “Have you been in a physical fight, assaulted, or deliberately hit anyone in the past 5 years’ and if they had carried a knife. They were asked about the outcome of violence, victims, number of incidents, location, gang fights, whether they were gang members, and used or carried weapons, previous criminal convictions and imprisonment. They were asked if they had been a victim of violence and feared assault, whether violence was instrumental (to obtain money, drugs or sex), and if they had deliberately looked for a fight, often ruminated about violence, found violence exciting, easily lost their temper, or became violent if disrespected.

Adverse Childhood Experiences (ACEs): They were asked if they had witnessed violence in their home, or been subjected to physical, sexual abuse, or neglect, had been in care, or had experienced a serious injury before age 16.

Life Events and Daily Living: Participants were asked whether they had a close relationship, had moved home in the preceding year, been separated or divorced, or fired, had experienced serious money problems, lacked educational qualifications, were not in education employment or training, or had experienced life-threatening injury, or homelessness. Finally, we asked whether friends encouraged them in criminality or drug taking and about their use of leisure time.

Socioeconomic deprivation was measured using a ranking on the Index of Multiple deprivation (IMDR) which measures the proportion of the population in an area experiencing deprivation according to domains of income, employment, health and disability, education skills and training, barriers to housing and services, crime, and living environment [25].

#### Psychiatric morbidity/substance misuse

The Psychosis Screening Questionnaire [26] covered 5 psychotic symptoms; participants screen positive for psychosis when ≥3 criteria are met. The Hospital Anxiety and Depression Scale [27] was used to define Anxiety and Depression based on scores of > 11 in the past week. Scores of 8–15 on the Alcohol Use Disorders Identification Test represent hazardous drinking, 16–19 a more severe state of alcohol abuse, and > 20 indicates need for clinical assessment for alcohol dependence [28]. These three AUDIT score ranges were mutually exclusive for participants. Scores > 25 on the Drug Use Identification Test indicated the participant screened positive for dependence on drugs. A score 6 or above usually indicates problems related to drug misuse. Scores 6–24 were classified a drug abuse [29].

Questions from the Structured Clinical Interview for DSM-IV Personality Disorders Screening Questionnaire [30] identified Antisocial Personality disorder (ASPD) when 3 or more of 7 items were present in adulthood and Conduct disorder when 3 or more of 15 items were present before age 15 years. For a diagnosis of ASPD both Conduct disorder and antisocial behaviour in adulthood are required.

#### Physical health/risks

We included a series of factors indicating current poor physical health or a risk of future physical health problems. These comprised self-reported physical health as poor or fair, taking medication for a physical illness, high fat/low fibre diet, lack of exercise, smoking, and height.

#### Sexual Health

Participants were questioned about their sexual behaviour, including whether they had had ten or more sexual partners in the past year, sex usually occurred when intoxicated, they rarely/ never used contraceptives, had had sex with a prostitute on ten or more occasions, sex with multiple partners during the same encounter, they were currently having sex with men, anal sex on any occasion. They were also asked whether they had forced partners to have sex against their will in more than half of encounters in the past year.

### Statistical analysis

Our analysis aimed to compare the characteristics of violence in GE and in the representative survey. We anticipated that violence prevalence would be higher in GE and carried out further analysis to investigate whether differences could be explained by poverty measured by socio-economic deprivation at small-area level (representing neighbourhood or community level of our ecological model) and unemployment (representing individual level). We further investigated exposures of adverse childhood experiences, daily living and stressful experiences in adulthood, and psychiatric morbidity (including substance misuse), physical and sexual health on violence as outcome. We adjusted our models for our proxy measure of poverty using socio-economic deprivation and unemployment. The survey primarily measured individual level variables and their associations and did not measure societal level factors. We used the extensive reviews that have been carried out by Public Health researchers in Scotland to contextualize our findings within large scale social forces that have impacted on men in Glasgow in areas characterised by high levels of socioeconomic deprivation and clusters of adverse health conditions, including GE.

Following data collection, weights were constructed for each survey using Random Iterative Method (RIM) weighting to ensure the representativeness of the sample to the population (weighted for non-response and quota sampling). All descriptive and subsequent statistical comparisons were based on weighted data. We used robust standard errors to account for clustering within postcodes. All analyses were performed using SPSS 26.0.

Frequencies were reported for demographic distributions among non-violent and violent men in surveys, with corresponding logistic regressions and linear regression for continuous variables (age and IMDR). Logistic regression analyses and Bonferroni correction were used to 1) compare the characteristics of violence among all men in both surveys before and after adjustments for socioeconomic deprivation and unemployment with all men in national survey as reference group and violent characteristic as outcome; 2) explore differences in risk factors (daily living, stressful experiences, peer group, employment, leisure and adverse childhood experiences) using non-violent men in national survey as reference group and violent men as outcome in multinomial logistic regression; and 3) compare the difference of the effect of violence on psychiatric morbidity, physical health/health risks and sexual health between violent men in national survey and GE, using non-violent men in national survey as reference group. Adjustments were made for age, unemployment and IMDR.

## Results

The weighted sample included 2665 men, aged 18–34 years: 1897 (71.4%) were in the national survey and 759 (28.6%) in GE. The total subgroup of 944 (35.5%) men who reported perpetrating violence in the past 5 years included 655 (34.5%) in the national survey and 289 (38.1%) in GE. Table 1 compares demography between survey samples and violent and non-violent men. Non-violent men in GE were more likely to be single, unemployed and unlikely to be non-UK born or of black and minority ethnic background. The same demographic characteristics were found for violent GE men compared to violent men in the representative survey, except that all were white.

**Table 1:** Demographic characteristics of survey samples and for non-violent and violent men (*n* = 2656)

	Non-violent men (***n*** = 1712, 64.5%)				Violent men (***n*** = 944, 35.5%)		
	National *N* = 1242 (46.8%)	Glasgow east *N* = 470 (17.7%)	National cf. Glasgow east		National *N* = 655 (24.7%)	Glasgow east *N* = 289 (10.9%)	National cf. Glasgow east
	N(%)	N(%)	AOR(95%CI)		N(%)	N(%)	AOR(95%CI)
Non-UK born	133(10.8)	11(2.4)	0.19***(0.10–0.36)	Non-UK born	40(6.2)	2(0.7)	0.10**(0.02–0.43)
Single	676(54.8)	326(69.7)	1.89***(1.51–2.37)	Single	437(67.2)	216(75.0)	1.45*(1.06–1.99)
Unemployed	335(27.6)	221(47.7)	2.40***(1.92–2.99)	Unemployed	263(41.2)	177(62.8)	2.41***(1.81–3.22)
*Ethnicity*				*Ethnicity*			
White	1075(86.7)	457(97.9)	7.26***(3.76–14.02)	White	600(91.5)	289(100.0)	/
Black	65(5.2)	3(0.6)	0.11***(0.03–0.36)	Black	25(3.8)	0	/
Asian	89(7.2)	7(1.5)	0.20***(0.09–0.43)	Asian	28(4.3)	0	/
Other	11(0.9)	0	/	Other	3(0.5)	0	/
	Mean(SD)	Mean(SD)	β(95%CI)		Mean(SD)	Mean(SD)	β(95%CI)
Age (years)	26.72(4.84)	26.97(5.42)	0.25(−0.28–0.79)	Age (years)	25.12(5.0)	26.06(5.53)	0.94**(0.23–1.65)
IMDR	12,774(9998)	1259(1424)	− 11515***(−12,493--10,607)	IMDR	11,847(9640)	1354(1475)	− 10493***(−11,612--9373)

Adjusted for age

### Comparisons of violent men

Criminality and Violence: Table 2 compares violence and associated criminality between men in the national survey and those from GE. More men in GE reported violence, gang fights, and fighting at sporting events, and more had used a weapon. There was no difference in the prevalence of those taking part in 5 or more violent altercations. More Glasgow men in general (and those who had been violent) had criminal convictions, been in prison and carried a knife. Violent Glasgow men were more likely to report their victims were family members, friends, or other known persons than violent men in the national survey. They were less likely to assault strangers, more likely to perpetrate violence in their own home or in other’s homes. There was no difference between GE and the rest of the sample in reporting violence in a bar/pub or outdoors. They were more likely to report victim injury, fear of violent victimisation, volatile temper, violence due to disrespect, regrets for violence, excitement from violence, seeking fights, and instrumental violence. After adjustment for unemployment and socioeconomic deprivation, violent men in GE and elsewhere were equally likely to report violence in the past 5 years, violence at sporting events, previous convictions for violence, drugs offences, theft/burglary, carrying a knife, violence towards family members, violence at home, victim injury, loss of temper, excitement from violence, and deliberately looking for fights.

**Table 2:** Comparison of Characteristics of Violence in National and Glasgow East Surveys (n = 2656)

	All men (n = 2656)							
	National *N* = 1897 (71.4%)		Glasgow east *N* = 759 (28.6%)		National cf. Glasgow east			
					Model 1		Model 2	
	N	%	N	%	OR	95%CI	OR	95%CI
**Characteristic of violence**								
Violence (past 5 years)	655	34.5	289	38.1	1.20*	1.00–1.43	0.98	0.80–1.21
Gang fight	56	2.9	67	8.8	3.32***	2.29–4.79	2.52***	1.58–4.03
Violence sports events	113	6.0	72	9.5	1.70***	1.24–2.32	1.37	0.94–2.00
5 or more fights	93	5.0	50	6.8	1.40	0.98–2.01	1.03	0.67–1.57
Used a weapon (ever)	141	7.6	120	15.9	2.30***	1.77–2.98	1.54*	1.11–2.13
**Associated criminality/violence**								
Previous Violence conviction	140	7.3	108	14.1	2.08***	1.59–2.71	1.28	0.92–1.78
Previous drugs	79	4.2	80	10.5	2.66***	1.93–3.68	1.42	0.94–2.13
Previous theft/burglary	85	4.5	66	8.7	1.97***	1.41–2.75	1.15	0.76–1.76
Carrying knife	106	5.5	84	11.0	2.14***	1.59–2.90	1.33	0.91–1.92
Gang member	20	1.1	12	1.6	1.49	0.72–3.08	1.28	0.53–3.13
Ever in prison	68	3.6	88	11.6	3.37***	2.43–4.70	1.89**	1.23–2.90
**Attitude towards Violence**								
Fear violent victimization	279	15.5	174	24.2	1.77***	1.43–2.20	1.69***	1.30–2.19
Easily lose temper	208	11.6	127	18.4	1.73***	1.36–2.20	1.30	0.97–1.74
Violence due to disrespect	345	20.6	213	34.1	2.03***	1.66–2.50	1.75***	1.36–2.24
Regretted violence	286	15.8	187	25.7	1.84***	1.50–2.27	1.44**	1.11–1.86
Excitement violence	118	6.4	68	9.2	1.51**	1.11–2.07	1.13	0.78–1.65
Deliberate fights	77	4.1	51	6.8	1.74**	1.21–2.51	1.34	0.86–2.08
Instrumental violence	141	7.6	120	15.9	2.30***	1.77–2.98	1.54**	1.11–2.13
**Victim of Violence**								
Intimate partner	156	8.4	73	9.7	1.13	0.85–1.52	0.76	0.54–1.07
Family member	71	3.8	52	6.9	1.90***	1.31–2.74	1.39	0.88–2.20
Friends	154	8.1	108	14.2	1.92***	1.47–2.49	1.64**	1.18–2.28
Known person	199	10.5	130	17.1	1.79***	1.41–2.28	1.56**	1.16–2.09
Stranger	349	18.5	120	15.8	0.84	0.67–1.06	0.78	0.60–1.02
Police	53	2.8	21	2.8	1.01	0.60–1.68	0.65	0.36–1.19
**Location**								
Home	78	4.1	60	7.9	1.99***	1.40–2.81	1.25	0.81–1.91
Others’ home	78	4.1	107	14.1	3.91***	2.88–5.31	2.32***	1.59–3.40
Street/Outside	377	19.9	155	20.4	1.04	0.85–1.29	0.94	0.73–1.20
Bar/pub	281	14.9	117	15.4	1.05	0.83–1.33	1.06	0.80–1.41
Others	31	1.6	11	1.4	0.77	0.49–1.20	0.72	0.43–1.19
**Outcome**								
Victim injury	307	16.2	158	20.8	1.37**	1.11–1.70	1.08	0.84–1.40
Perpetrator injury	82	4.3	23	3.0	0.67	0.42–1.08	0.59	0.34–1.02
Police called	185	9.8	73	9.6	0.98	0.74–1.31	0.73	0.52–1.02

Model 1: Adjusted for age

Model 2: Adjusted for age, unemployment, IMDR

### Comparison of violent versus non-violent men

Adverse childhood experiences: Table 3 shows that non-violent men in GE were more likely to have witnessed violence in the home during childhood but with no overall differences in experiences of childhood adversity. Violent men in both the national and GE surveys reported more of all individual experiences of childhood adversity except sexual abuse compared to non-violent men in the national survey. When comparing violent men, those in GE were more likely to report witnessing violence in the home and less likely to report no adverse childhood experiences. However, following adjustment for unemployment and IMDR rank these differences were no longer significant.

**Table 3:** Comparisons of childhood adverse experiences in non-violent and violent men in surveys (n = 2656)

Adverse Childhood experiences	Non-Violent men			Violent men				Violent men	
	National N = 1242 (46.8%)	Glasgow east *N* = 470 (17.7%)		National *N* = 655 (69.4%)		Glasgow east *N* = 289 (30.6%)		National cf. Glasgow East	
	N(%)	N(%)	Model 1 AOR(95%CI)	N(%)	Model 1 AOR(95%CI)	N(%)	Model 1 AOR(95%CI)	Model 1 AOR(95%CI)	Model 2 AOR(95%CI)^a^
Bullying	341(27.5)	116(24.7)	0.86 (0.68–1.10)	249(38.0)	1.67*** (1.37–2.05)	98(33.9)	1.46* (1.04–1.79)	0.86 (0.68–1.10)	0.86 (0.61–1.21)
Witnessed violence in home	85(6.8)	51(10.9)	1.63** (1.13–2.35)	131(20.0)	3.64*** (2.71–4.89)	100(34.6)	7.43*** (5.34–10.32)	1.62* (1.12–2.34)	1.36 (0.93–1.98)
Sexual abuse	27(2.2)	6(1.3)	0.55 (0.22–1.37)	21(3.2)	1.65 (0.92–2.95)	7(2.4)	1.10 (0.47–2.60)	0.55 (0.22–1.36)	0.44 (0.16–1.19)
Physical abuse	51(4.1)	16(3.4)	0.82 (0.46–1.44)	71(10.8)	3.02*** (2.07–4.40)	39(13.5)	3.71*** (2.39–5.75)	0.82 (0.46–1.44)	0.84 (0.51–1.38)
Neglect	27(2.2)	13(2.8)	1.22 (0.62–2.39)	49(7.5)	3.82*** (2.36–6.18)	23(8.0)	3.91*** (2.21–6.93)	1.23 (0.63–2.41)	1.02 (0.54–1.93)
Serious injury	17(1.4)	11(2.3)	1.75 (0.82–3.73)	30(4.6)	3.68*** (2.01–6.74)	19(6.6)	5.24*** (2.70–10.17)	1.74 (0.82–3.71)	1.42 (0.67–2.99)
In care	22(1.8)	11(2.4)	1.31 (0.62–2.73)	49(7.7)	4.78*** (2.85–8.03)	26(9.2)	5.61*** (3.12–10.09)	1.31 (0.63–2.75)	0.79 (0.44–1.44)
No. of ACE									
0	837(67.4)	302(64.3)	0.87 (0.70–1.09)	311(47.5)	0.42*** (0.35–0.51)	112(38.8)	0.30*** (0.23–0.39)	0.71* (0.54–0.94)	0.83 (0.59–1.16)
4 or more	29(2.3)	10(2.1)	0.88 (0.43–1.83)	42(6.4)	3.23*** (1.99–5.26)	28(9.7)	4.63*** (2.70–7.93)	1.42 (0.86–2.36)	1.09 (0.59–2.01)
	Mean(SD)	Mean(SD)	*P* value	Mean(SD)	P value	Mean(SD)	P value	P value	P value
No of ACEs	0.52(1.01)	0.55(0.96)	0.811	1.04(1.37)	< 0.001	1.26(1.47)	< 0.001	0.066	0.066

ACE: adverse childhood experiences

Model 1 Adjusted for age

Model 2 Adjusted for age, unemployment and IMDR

Adversity, Leisure and Education: Table 4 shows that non-violent men in GE were unlikely to have a close relationship, lived alone, more likely to experience homelessness, separation, had friends who encouraged them in crime and to take drugs, had no educational qualifications and were not in education, employment or training (NEET) and their social lives involved sporting events such as football. Violent men in GE showed many similarities in daily living similar to non-violent men in the same location. Violent men in GE were more likely to live alone, while their counterparts in the national survey were more likely to have moved home. Leisure patterns also differed between sub-groups of violent men. Those in the national survey were more likely to spend two or more nights a week partying and attending clubs/discos; those in GE more likely to attend sporting events and spend time in snooker/pool halls. Following adjustment for unemployment and socioeconomic deprivation, violent men in Glasgow were still less likely to have a close relationship, less likely to have moved home, more likely to be separated, have friends who encouraged drug misuse, NEET, and spend their leisure time attending sports events and snooker/pool halls.

**Table 4:** Comparisons of Daily Living and Stressful Experiences among Non-Violent and Violent men (n = 2656)

	Non-Violent men			Violent men				Violent men	
	National N = 1242 (46.8%)	Glasgow east N = 470 (17.7%)		National N = 655 (69.4%)		Glasgow east N = 289 (30.6%)		National cf. Glasgow East	
	N(%)	N(%)	Model 1 AOR(95%CI)	N(%)	Model 1 AOR(95%CI)	N(%)	Model 1 AOR(95%CI)	Model 1 AOR(95%CI)	Model 2 AOR(95%CI)^a^
**Daily Living**									
Close relationship	763(65.1)	234(50.9)	0.53*** (0.43–0.67)	362(57.4)	0.81* (0.66–0.99)	131(45.8)	0.47*** (0.36–0.61)	0.59*** (0.44–0.79)	0.54*** (0.39–0.77)
Lives alone	173(13.9)	129(27.4)	2.31*** (1.77–3.00)	94(14.4)	1.21 (0.92–1.60)	71(24.6)	2.14*** (1.56–2.95)	1.74** (1.21–2.48)	1.17 (0.75–1.80)
Moved home past year	337(27.6)	67(14.6)	0.45*** (0.33–0.60)	222(34.2)	1.34** (1.09–1.65)	68(23.7)	0.81 (0.60–1.09)	0.59*** (0.43–0.82)	0.49*** (0.34–0.71)
**Stressful Experiences**									
Homelessness	45(3.6)	35(7.4)	2.12*** (1.34–3.35)	102(15.6)	5.43*** (3.75–7.86)	67(23.2)	8.42*** (5.60–12.66)	1.54* (1.09–2.19)	1.06 (0.69–1.62)
Separation	75(6.0)	48(10.2)	1.71** (1.17–2.52)	64(9.8)	2.04*** (1.43–2.90)	42(14.5)	2.81*** (1.86–4.23)	1.40 (0.91–2.14)	1.08* (1.02–3.02)
Serious money problems	137(11.0)	61(13.0)	1.15 (0.83–1.60)	191(29.2)	3.98*** (3.09–5.12)	94(32.5)	4.27*** (3.13–5.82)	1.07 (0.78–1.45)	0.91 (0.63–1.32)
**Peer group**									
Friends encourage crime	32(2.7)	32(7.1)	2.77*** (1.67–4.58)	130(21.5)	9.63*** (6.43–14.41)	70(25.8)	12.35*** (7.91–19.28)	1.28 (0.91–1.79)	1.09 (0.73–1.64)
Friends encourage drugs	160(12.9)	79(16.8)	1.37* (1.02–1.84)	250(38.2)	4.03*** (3.20–5.08)	149(51.6)	7.17*** (5.39–9.54)	1.78*** (1.34–2.35)	1.67** (1.19–2.34)
**Employment**									
No educational qualification	101(8.1)	90(19.1)	2.63*** (1.93–3.58)	100(15.3)	2.19*** (1.63–2.95)	81(28.0)	4.58*** (3.29–6.36)	2.09*** (1.50–2.93)	1.25(0.82–1.89)
NEET	149(12.4)	148(32.8)	3.45*** (2.66–4.48)	128(20.5)	1.83*** (1.41–2.38)	148(52.3)	7.78*** (5.82–10.40)	4.21*** (3.10–5.70)	4.21*** (2.44–7.25)
**Leisure**									
Partying	137(11.4)	29(6.3)	0.51** (0.34–0.78)	118(18.5)	1.50**(1.14–1.98)	44(15.5)	1.32 (0.91–1.92)	0.89 (0.60–1.31)	0.87 (0.55–1.37)
Sports events	552(45.5)	262(56.6)	1.59*** (1.28–1.97)	323(51.5)	1.20 (0.98–1.45)	176(61.8)	1.89*** (1.45–2.47)	1.58** (1.18–2.11)	1.66** (1.18–2.35)
Club/discos	144(11.6)	45(9.6)	0.79 (0.55–1.14)	137(20.9)	1.73*** (1.33–2.24)	42(14.5)	1.17 (0.80–1.71)	0.68* (0.46–1.00)	0.66 (0.42–1.03)
Visit snooker/pool halls	69(5.7)	31(6.7)	1.17 (0.75–1.81)	54(8.7)	1.40 (0.96–2.03)	44(15.5)	2.88*** (1.92–4.32)	2.07*** (1.35–3.18)	2.40** (1.39–4.16)

Model 1 Adjusted for age; Model 2 adjusted for age, unemployment, IMDR

Psychiatric Morbidity, Physical and Sexual Health: Table 5. Shows that non-violent men in GE were more likely to drink hazardously, abuse drugs and were drug-dependent, had poor physical health, long-standing physical conditions, smoked heavily, and although sex usually took place when intoxicated they were less likely to report anal sex, rare use of contraceptives, sex with more than one person, and sex with men. Compared to non-violent men in the national survey, violent men in the national survey and in GE were similar in GE, were being more likely to show all categories of psychopathology except depression, most categories of physical health/risks, and poor sexual health. Violent men in the national survey were less likely to report lack of exercise. Violent men in GE were less likely to report sex with men.

**Table 5:** Comparison of psychiatric morbidity, physical health, and sexual health of Non-Violent and Violent Men in surveys (n = 2656)

	Non-Violent men			Violent men				Violent men	
	National N = 1242 (46.8%)	Glasgow east N = 470 (17.7%)		National N = 655 (69.4%)		Glasgow east N = 289 (30.6%)		National cf. Glasgow East	
**Psychiatric Morbidity**	N(%)	N(%)	Model 1 AOR(95%CI)	N(%)	Model 1 AOR(95%CI)	N(%)	Model 1 AOR(95%CI)	Model 1 AOR(95%CI)	Model 2 AOR(95%CI)^a^
Psychosis (PSQ ≥ 3)	9(0.7)	6(1.3)	1.97 (0.70–5.53)	28(4.4)	6.90*** (3.18–14.97)	20(7.0)	10.90*** (4.83–24.58)	1.58 (0.87–2.87)	0.92 (0.46–1.85)
Anxiety^#^	85(6.9)	42(9.1)	1.26 (0.84–1.89)	115(17.8)	3.29*** (2.40–4.52)	64(22.2)	3.96*** (2.71–5.79)	1.20 (0.83–1.73)	1.08 (0.68–1.70)
Depression^#^	82(6.7)	35(7.6)	1.05 (0.68–1.63)	51(7.9)	0.78 (0.52–1.16)	29(10.1)	0.94 (0.58–1.52)	1.19 (0.71–2.00)	0.95 (0.50–1.79)
Hazardous drinking	328(27.4)	173(39.1)	1.71*** (1.36–2.15)	249(41.1)	1.78*** (1.45–2.19)	106(39.8)	1.72*** (1.30–2.26)	0.96 (0.72–1.29)	0.96 (0.67–1.36)
Alcohol abuse	63(5.3)	18(4.1)	0.78 (0.46–1.33)	64(10.6)	2.09*** (1.45–3.01)	24(9.0)	1.73* (1.05–2.83)	0.84 (0.51–1.38)	0.86 (0.48–1.55)
Alcohol dependence	54(4.4)	25(5.4)	1.22 (0.75–1.98)	77(12.1)	3.02*** (2.10–4.35)	59(21.4)	5.92*** (3.98–8.80)	1.98*** (1.36–2.87)	1.70* (1.06–2.71)
Drug abuse	84(7.0)	48(10.9)	1.64* (1.13–2.37)	130(21.3)	3.52*** (2.62–4.73)	72(28.6)	5.27*** (3.71–7.50)	1.48* (1.06–2.07)	1.37 (0.91–2.06)
Drug dependence	7(0.6)	9(1.9)	3.51* (1.28–9.58)	34(5.3)	11.70*** (5.07–26.99)	38(13.5)	30.28*** (13.16–69.71)	2.62*** (1.61–4.27)	1.94* (1.04–3.61)
Antisocial personality disorder	66(5.4)	16(3.4)	0.61 (0.35–1.07)	192(30.8)	8.41*** (6.20–11.40)	119(42.2)	13.32*** (9.43–18.82)	1.60** (1.19–2.14)	1.20 (0.85–1.71)
Suicide attempt	43(3.5)	13(2.8)	0.77 (0.41–1.45)	70(11.0)	3.59*** (2.41–5.35)	34(12.0)	3.88*** (2.42–6.21)	1.09 (0.70–1.68)	0.78 (0.46–1.32)
**Physical health**									
Poor/fair	119(9.7)	83(17.8)	1.98*** (1.46–2.69)	90(14.0)	1.76** (1.27–2.29)	80(27.8)	3.83*** (2.77–5.30)	2.24*** (1.58–3.16)	1.87** (1.21–2.88)
Longstanding condition	61(4.9)	41(8.8)	1.77** (1.17–2.69)	52(8.0)	2.02*** (1.36–2.98)	53(18.6)	4.81*** (3.21–7.19)	2.38*** (1.56–3.64)	1.09 (0.67–1.78)
Serious/life threatening injury	21(1.7)	7(1.5)	0.82 (0.34–1.97)	31(4.7)	3.30*** (1.87–5.83)	28(9.7)	6.62*** (3.68–11.89)	2.00* (1.17–3.42)	2.10* (1.03–4.27)
High fat/low fibre diet	70(5.6)	31(6.6)	1.22 (0.79–1.87)	51(7.8)	1.32 (0.93–1.88)	43(14.9)	2.81*** (1.88–4.20)	2.18*** (1.41–3.37)	2.10** (1.21–3.64)
Lack of exercise	136(11.0)	54(11.5)	1.04 (0.75–1.46)	47(7.2)	0.66* (0.46–0.93)	39(13.5)	1.30 (0.89–1.90)	2.00** (1.27–3.13)	1.41 (0.80–2.51)
Smoking ≥15/day	136(11.0)	87(18.5)	2.13*** (1.58–2.86)	77(12.0)	2.89*** (2.31–3.63)	62(21.5)	3.95*** (2.89–5.41)	2.02*** (1.40–2.92)	2.35*** (1.47–3.74)
Obesity	146(13.6)	53(12.3)	0.87 (0.62–1.22)	61(11.0)	0.90 (0.65–1.24)	32(12.4)	0.93 (0.61–1.40)	1.08 (0.68–1.72)	0.92 (0.54–1.58)
	Mean(SD)	Mean(SD)	β(95%CI)	Mean(SD)	β(95%CI)	Mean(SD)	β (95%CI)	Model 1 β (95%CI)	Model 2 β (95%CI)
Height (cm)	175.9(7.4)	174.6(7.3)	−1.29** (−2.12- -0.45)	177.0(7.7)	1.13** (0.36–1.90)	173.8(8.1)	−2.06*** (−3.07- -1.04)	−3.25*** (−4.40- -2.11)	− 2.74*** (−4.11- -1.37)
**Sexual health**		N(%)	AOR(95%CI)	N(%)	AOR(95%CI)	N(%)	AOR(95%CI)	Model 1 AOR(95%CI)	Model 2 AOR(95%CI)
STI	75(6.4)	38(8.4)	1.34 (0.89–2.01)	108(17.4)	3.17*** (2.31–4.35)	56(19.8)	3.68*** (2.53–5.35)	1.17 (0.82–1.67)	1.06 (0.69–1.62)
Anal sex	235(20.1)	37(8.0)	0.34*** (0.24–0.49)	184(30.2)	1.82*** (1.45–2.28)	65(23.0)	1.22 (0.89–1.66)	0.68* (0.49–0.94)	0.73 (0.49–1.07)
≥ 10 sex partners (past year)	27(2.4)	16(3.6)	1.49 (0.80–2.80)	48(7.8)	3.11*** (1.92–5.05)	19(6.9)	2.86*** (1.56–5.22)	0.94 (0.54–1.63)	0.87 (0.45–1.66)
Coercive sex	27(2.4)	5(1.2)	0.51 (0.20–1.28)	46(7.8)	3.25*** (1.99–5.30)	6(2.5)	0.94 (0.38–2.35)	0.30** (0.12–0.71)	0.25** (0.10–0.64)
Contraceptive sex rare/never	282(25.5)	77(17.4)	0.62** (0.47–0.82)	178(29.1)	1.24 (0.99–1.55)	59(20.9)	0.79 (0.57–1.08)	0.64** (0.46–0.89)	0.51*** (0.35–0.76)
≥ 10 prostitutes (ever)	67(5.4)	24(5.1)	0.94 (0.58–1.52)	58(8.9)	1.83*** (1.27–2.65)	28(9.7)	1.91** (1.20–3.04)	1.04 (0.64–1.68)	1.18 (0.65–2.14)
Sex usually intoxicated	145(13.1)	76(20.1)	1.69*** (1.24–2.29)	173(29.0)	2.61*** (2.03–3.35)	131(53.5)	7.42*** (5.46–10.08)	2.84*** (2.09–3.87)	2.84*** (1.95–4.15)
Sex > 1 person	185(15.8)	43(9.3)	0.54** (0.38–0.76)	231(37.1)	3.31*** (2.63–4.17)	87(30.5)	2.41*** (1.79–3.25)	0.73* (0.54–0.98)	0.71 (0.50–1.02)
Sex with men	67(5.6)	8(1.7)	0.31** (0.15–0.64)	30(4.7)	0.84 (0.54–1.31)	2(0.7)	0.11** (0.02–0.47)	0.13** (0.03–0.60)	0.14* (0.03–0.65)

Model 1 Adjusted for age

Model 2 Adjusted for age, unemployment, IMDR

# Adjusted for anxiety or depression

We next compared violent men in the national survey and GE. Alcohol, drug abuse, drug dependence and ASPD, poor/fair physical health, longstanding health conditions, serious life-threatening injuries, high-fat/low fibre diet, lack of exercise, short stature, and sex usually when intoxicated were all more prevalent among violent men in GE than violent men in the national survey. But anal sex, coercive sex, rare contraceptive use, sex with more than one person in same encounter, and sex with men were less prevalent. When we adjusted these differences among violent men for socioeconomic deprivation and unemployment, alcohol and drug dependence, poor/fair health, serious life-threatening injury, high fat/low fibre diet, heavy smoking, short stature and sex usually when intoxicated remained significantly more prevalent. Reported sexual health-related behaviours of coercive sex, rare contraceptive use, and sex with men were significantly less prevalent in violent GE men.

## Discussion

Our study confirmed that violent men in Britain, and GE in particular, experience multiple adverse exposures over the life-course. In adulthood, markers of inequality among violent men included an aggregation of inter-related physical and sexual health conditions, together with psychiatric morbidity, where the odds of association were greater for many of these factors among violent Glaswegian men than violent men in the general population. The excess of violence and multiple associated health conditions was partly explained by higher levels of unemployment and socioeconomic deprivation in GE. However, several key features could not be explained by these factors and were influenced by other effects specific to GE. Drug and alcohol dependence were among the most characteristic features of Glasgow men – whether violent or not – and appeared to be strongly influenced by factors within the interpersonal level of the ecological model that were more prevalent in GE. It is possible that substance misuse had resulted in few close relationships or friendships in these men’s lives except other drug and alcohol misusers, those who encouraged them in criminality, and with social isolation a consequence for many due to inability to form stable relationships. At the community level (and because GE is a relatively small and relatively isolated area of Glasgow in terms of public transport and access to facilities), our findings indicate a social environment with a high proportion of drug and alcohol-abusing men with no occupation, unlikely to move away to seek employment elsewhere, who are physically unhealthy, involved in criminality, and where violence is used to facilitate their criminality but also to command respect. The highly stressful nature of this lifestyle in GE was indicated by a quarter of violent men themselves in fear of becoming victims of violence.

### Violence in Glasgow east

Although GE showed a small overall excess of men reporting violence, their violence showed specific, qualitative differences, including instrumental violence within a criminal lifestyle where crime was viewed not as an isolated incident but a lifelong commitment [31], with weapon use and group violence. Two in every five young adult men in GE screened positive for ASPD, indicating a diagnosis of conduct disorder before age 15 years and an antisocial lifestyle in adulthood. Glasgow men also differed in their attitudes towards violence embedded within a specific subculture partly shared by non-violent men living in the same area. Violence at sporting events partly reflected traditional support for rival football teams in Glasgow, divided according to religious affiliation and sectarianism, but also where amateur games between rival groups resulted in outbreaks of group violence [32].

Although relatively few reported gang membership, more had been involved in gang fights. At the time of the survey, men in Glasgow were increasingly under police pressure from a violence reduction strategy targeting violence and gangs [33]. Our findings may reflect successful police intervention and the recent decline in violence in Scotland has been attributed to this programme. Alternatively, violence and the structure of gangs may have already changed by the time of the survey for other reasons, with loosely structured groups of young men still fighting together, but no longer adhering to the self-identified street gangs observed a decade earlier [3]. Fraser [32] suggested that a process of evolution of street gangs from previous claiming and defending neighbourhood territory and fighting with other rival gangs into more organized crime has not taken place in Glasgow, contrasting with evolutionary change in patterns of gang activity observed in London [34]. Possible explanations for decline of gang identity also included less attention from the media as a source of pride for young men in areas of the city such as GE, changes in use of leisure time with less time spent on the street, changes to work and a working class identity (previously associated with a macho, violent self-presentation) having become irrelevant in the context of no opportunities for work in heavy industry, and changes in policing. However, since our survey, gangs in Glasgow have shown increasing similarities to recent patterns observed in England, becoming criminal groups involved in drug distribution and supply as entry into the global drugs market becomes more readily accessible [3]. Within this changing context, the function of violence also changes because the primary focus is supporting a business model where violence is minimized because it draws unwanted attention from the police, who may disrupt business, but where it may become necessary to protect supplies and profits, together with intimidation or removal of rivals who are in market competition [35].

Victims of violent men in GE were more commonly their family members (likely to increase their social isolation), friends or acquaintances, whereas in the national survey victims were usually strangers (although there was no significant difference in prevalence of stranger victims in both surveys). Because GE is a small, socially isolated, urban area, inhabitants are unlikely to move and more likely to know each other. In both violent samples, violence occurred in the street and in bars/pubs, However, in GE it also occurred in their own or another’s home and witnessing violence in the home during childhood characterised the early lives of both violent and non-violent men in GE. In adulthood, this might have reflected altercations over drugs or groups of young men meeting to drink heavily together. Injuries to victims reflected their greater prevalence of weapon carrying and group violence.

### Ecological model

Our findings can be integrated in an ecological model of violence [19] in which complex, inter-related exposures result both in violence and in the aggregation of multiple, inter-related, adverse health outcomes (see Fig. 1). These systems exhibit both multi-finality (where the same factors lead to a range of mental and physical health outcomes as well as violence) and equi-finality (where diverse causes can produce the same disorder [36, 37]. This is shown in the adverse childhood experiences and adult outcomes we measured [38]. Moreover, many relationships between psychiatric, physical, sexual health, and violent outcomes in the ecological model are bi-directional. For example, men carried knives for protection and were consequently more likely to become involved in violence, thereby maintaining high levels of fear and anxiety. Injuries increased fear but additionally contributed to poor physical health and substance misuse to cope with anxiety. Drug misuse led in some cases to violence over drug deals. Drug and alcohol misuse further contributed to poor physical and mental health and high-risk sexual behaviour through multiple pathways of association. Our statistical strategy therefore follows that used in most studies of violence, particularly in psychiatry, where is it assumed that exposures such as substance misuse and severe mental illness lead to violence and where an aim of investigation is to establish a causal pathway to violence [39, 40]. However, our findings also reveal the severe limitations of our own and this conventional approach and where situational factors may have equal or greater impact on violence as outcome.

#### Individual level factors

Psychotic experiences are associated with violence, explained primarily by paranoid ideation [22]. Although depression has been linked to violence [39], this association was confounded by anxiety disorder, both in the present study and previously [22]. Drug dependence showed the strongest associations with violence, followed by ASPD and alcohol dependence. A similar pattern of association was observed in a British national survey [24]. However, the association between drug misuse and violence is highly complex. Different pathways need to be considered, including drug misuse leading to violence, violence leading to drug misuse, and both having common causes where the association is spurious [41].

Multiple risk factors for future physical ill-health within a lifestyle encouraging both violence and substance misuse may partly explain the shortened life expectancy in GE. Cohort studies show that mental health, substance misuse and behavioural problems in early adulthood set in motion the development of a lifelong cascade of physical health, including liver, cardiovascular, and cerebrovascular diseases and dementias. These have greater impact on persons of lower socioeconomic status [42].

Height conveys an advantage to men in violent altercations and violent men in Britain are generally taller. The association with short stature in GE was of considerable interest and could result from impact of aggregated adverse factors over successive generations. Although 80% of height variation is thought to be under genetic control in developed countries [43], global studies identify stunted growth as an important complex marker of environmental inequalities [44] and in GE could indicate a marker for men exposed to poorer nutrition and greater environmental stress in infancy and additional adverse exposures in utero.

The relationship between violence and sexual health has rarely been studied. However, its importance is increasingly recognised, firstly, due to high levels of sexual risk-taking behaviour and sexual assault of women by violent men and gang members [45], and secondly, among population subgroups subject to high levels of incarceration, as in GE. This can lead to changes in population male-female ratios. Men returning to the community on release have limited social and economic activity, often experience homelessness, poverty, unstable living, and vulnerability to STIs. They are more likely to have relationships with high-risk partners, with further impact from substance misuse [46, 47]. Risk-taking by violent men in GE corresponded to violent men in the national survey, with sex involving more sexual partners, including sex workers, sex with more than one person in the same encounter, and sex when intoxicated, resulting in higher prevalence of STIs. However, they were less likely to report sex with men and contrasted with violent men in the National survey in fewer reporting they had engaged in coercive sex with unwilling partners. The meaning of these findings require further investigation in the context of men in GE reporting fewer close relationships. It is possible that denial of homosexual activity corresponded to homophobic attitudes within a macho subculture, but would require further study.

#### Interpersonal level factors

Maltreatment and other adverse events in childhood were no more prevalent among violent men in GE than violent men in the national survey, except for witnessing violence in the home which has been shown to have a particularly important effect on future internalizing and externalising problems [48]. ACEs were key influences in increasing future risks of adverse physical and mental health outcomes across both samples [38, 49]. However, in GE, early adult experiences of encouragement from peers to use drugs, weak family ties, and a lack of close, supportive relationships may have interacted with childhood vulnerabilities to further increase the risk of violence and poor health in multiple domains in adulthood.

#### Community level factors

Community factors include area-level effects of socio-economic deprivation, which was exceptionally high in GE. For example, studies of intimate partner victimization of women and men in England and Wales have shown that community measures of social housing tenure, low household income, poor educational attainment, low social class and living in a multiply deprived area are all correlates [50]. Community factors in GE included living in an area of low social capital, where a high proportion of men had no occupation and were involved in criminality, living in a geographically isolated area (in terms of transport links in the city from employment opportunities), which in turn increased their social isolation and exclusion. Leisure opportunities were also more limited for men in GE than for other violent men, where football and going to pool halls appeared the predominant activities. Violent men elsewhere appeared more mobile. Those from GE tended to remain there, many living alone, in poor quality, local authority housing, without employment, education or training. The importance of the inter-relationship between different levels in the ecological model can be seen studies of neighbourhood crime and mental health in Scotland, where local crime is an important predictor, independent of individual and other contextual risk factors [51]. Nevertheless, previous research has not uniformly confirmed a simple correlation between levels of socio-economic deprivation and violence at area level and our findings suggest it was these additional social and physical environmental exposures in GE that had additional impact together with poverty at the community and individual level. For example, studies of IPV in developing countries have found that women living in the middle range of a socioeconomic scale were more likely to be victims [52–54] and that interpersonal level factors of men’s gender conservative attitudes, controlling behaviour, multiple sexual partnerships, and excessive alcohol use in the areas studied were more important than area-level poverty. Furthermore, studies of protective factors which buffer individuals and communities living in poverty demonstrate that violence is not an inevitable consequence of deprivation in the context of a stable economy, positive social norms, abundant resources, high levels of social cohesion, family support and rewards for prosocial community involvement [55, 56]. However, there were few indicators of these protective factors operating in GE.

Corresponding to community-level crime, exposure to community violence itself is a critical psychosocial stressor in many urban communities, impacting on health, particularly among children and adolescents [38]; mediating health impacts of poverty through direct psychosocial impacts, behaviour or susceptibility to other exposures [57]; and, consequently, playing a larger role in stress than previously understood. Studies have validated associations between community violence and perceived stress [58]. Furthermore, a focus groups study in New York found that community residents prioritised violence above all other community stressors [59]. J Cowley, J Kiely and D Collins [60] have proposed that an accumulation of multiple life stressors predispose socially disadvantaged persons in Glasgow to a range of health disorders and health-related behaviours. The burden of this chronic stress is accompanied by culturally promoted changes in personal behaviours: such as increased incidence of smoking, disordered eating and drinking. Stress-inducing lifestyle behaviours, in turn, drive other stress-elevating conditions, such as poor quality sleep, increased body mass index, reduced energy levels and reduced tendencies to engage in health-promoting physical activity behaviours [61]. These factors interact in a downward spiral, adding momentum to an insidious vicious cycle of self-perpetuating stress, whilst over-activation of the stress response simultaneously erodes stress resilience [60].

#### Societal level factors

Societal factors were not included in the study. However, large-scale political, economic, and cultural forces can give rise to clustered epidemics of various diseases and health-related behaviours which can occur in situations of changing political and economic conditions, shifting ecological and environmental conditions, and altering demographics and social behaviours [17]. Effects of societal factors of specific relevance to Glasgow have previously been reviewed by Public Health researchers in Scotland. Glasgow had shown progressive post-industrial decline during the twentieth century and by mid-twentieth century tenement slums were increasingly replaced by a new generation of post-war high-rise housing and large suburban housing estates, with population displacement. These are thought to have broken up long established community relationships and social structures through population displacement and were later identified as having concentrations of deprivation and criminality associated with loss of social capital. There was thought to be lower social capital in Glasgow than cities such as Manchester, Liverpool and Belfast despite similar socioeconomic deprivation in parts of those cities and post-industrialisation [6]. The 1970s and 1980s were characterised by a period of serious and accelerated decline in industry leading to mass unemployment and urban decay and the association between Glasgow and its reputation for youth gangs and high levels of violence was established during this period. Political policies and social changes beyond the control of the local population in deprived areas of Glasgow had included relocation of younger, skilled workers to “New Towns” away from the “declining city”, leaving concentrations of persons with few work skills and aspirations, and with these populations displaced to poor quality peripheral housing estates (as typified by the built environment of GE), often surrounded by a poor quality physical environment with vacant and derelict land (where fights between youth gangs would typically occur), concentrations of high-risk subgroups with mental illness, addictions and former homeless people relocated to hostels and cheaper accommodation in these areas, and a ‘democratic deficit’ with experiences of despondency, disempowerment, and lack of sense of control [6].

### Limitations

Our study has several limitations. Our hypothesis that an aggregation of physical, sexual and psychiatric risk factors explained high levels of violence in Glasgow and other deprived areas of Scotland is based on results from a single geographical location (GE). However, there are other Scottish inner-urban areas with similar levels of socioeconomic deprivation in which our findings may be replicated. In addition, because of the cross-sectional nature of our study, we could not determine the direction of many of our associations. This is highly important because our method selected variables from domains of psychiatric morbidity, physical ill-health, and sexual health as exposures with violence as outcome, corresponding to many studies, particularly in psychiatry [62]. However, our findings suggest that these factors are bi-directional. Furthermore, that reverse causation cannot be ruled out.

Our survey was restricted to young adult men. Although women would be unlikely to show similar levels of violence, it would be of considerable importance to investigate differences in women’s violent behaviour in GE compared to elsewhere, together with other important differences such as health, childcare, substance misuse, and reasons for frequent breakdowns in relationships.

Our measure of violence was restricted to self-report and is likely to include both under- and over-estimates. However, criminal records data gives no indication of victim, location, whether others were involved, and introduces biases against sub-groups who are disproportionately arrested, as in GE. We did not interview participants to confirm diagnostic categories and relied on self-report. However, self-report can compare favourably with clinician assessments [63].

We used Random location quota sampling but do not have detailed information on the numbers who declined to participate in this survey. In most conventional survey methods, simple random sampling is used without replacement of participants who decline to participate and it is essential to know the numbers who actually declined and their demographic characteristics. Because declining to participate in a survey is not random in a population, and men, younger persons, and those of lower social status in the UK are more likely to decline, it is essential to include sufficient cases matching these characteristics, particularly where variables such as violence and criminal activity are a key area of investigation. In a more representative survey aimed to cover a wider age range, including men and women, a large and expensive survey is necessary to capture sufficient numbers of this subgroup. Furthermore, if attrition is sufficiently large, statistical weighting may still not compensate for many missing individuals who declined from a particularly hard-to-reach subgroup. The quota sampling method is based on the National Census, participants are identified and included according to representative strata and their actual frequency in the population, and the surveying organization must ensure their workforce meet a representative quota based on the census. Declining to participate means that another individual will be approached within the same area and matching the same demographic characteristics of each individual who declined until an agreement is secured to participate and the quota is filled. This method means that the number who declined does not matter in terms of attrition in the survey because the survey has matched a representative subgroup of the population based on the census. However, because the characteristics of those who decline are unknown it is still possible that the individuals who agreed to participate have differing qualitative characteristics, which remain unknown, despite matching the same demographic characteristics and coming from the same area (see Supplementary file).

### Implications

Violence should in future be investigated within a wider framework of multiple, interlinked health conditions and health-related behaviours. Previous studies of psychiatric morbidity and violence have tended to focus narrowly on associations with a limited range of psychiatric conditions rather than viewing violence as a public health problem within a wider aggregation of multiple health risks. Studies limited to a single diagnosis, a restricted range of environmental factors, and not taking into account potential area-level effects, cannot indicate the relevant public health interventions necessary to reduce violence. Our findings also indicate that future interventions should not narrowly focus on violence. Many risk factors associated with this aggregation of violence and physical, sexual and psychiatric morbidity were explained by greater socioeconomic deprivation and unemployment in GE. However, specific area-level effects included a sub-culture in which violence and attitudes to violence are condoned, together with an exceptional level of substance misuse in both violent and non-violent men.

Men in GE have experienced life-long, adverse interpersonal relationships where adverse childhood experiences are followed by negative peer influences within a socially isolated and excluded community. This leads to accumulative, multi-source stress [60] associated with educational failure, unemployment, inability to sustain intimate relationships, normalization of criminality and substance misuse, incarceration, group violence, and use of weapons in a subculture which promotes violence. These inequalities in physical and mental health are associated with considerably reduced life-expectancy [42] as well as violence.

A variety of programmes in USA, Scotland and elsewhere have taken a public health approach to reducing violence. Key characteristics of these programmes are an emphasis on the collaborative working between multiple sectors, partnership with communities/residents affected by violence, and close attention to accurate data collection and evaluation, including towards greater equity in outcomes. This approach is not solely focussed on law enforcement and improved outcomes in terms of violence reduction are seen as depending on partnerships across a number of sectors, for example education, health, social services, housing, youth services, and victim services [64]. The fall in violent convictions is largely attributed to the Violence Reduction Unit of Police Scotland, established in 2005 at a time of rising homicide rates [65]. The unit independently adopted a “Public Health” approach which has now become the dominant model for community violence reduction in the UK. However, it did not receive input from Public Health Agencies and the model was based on a successful programme implemented by police and social services in the USA [66] which was led by Police Scotland. Its aims were to reduce violence by working with health, education and social work agencies to achieve societal and individual attitudinal change by focusing on enforcement and contain and manage individuals who carry weapons and are involved in violent behaviour. Emphasis on enforcement was balanced by a rehabilitative approach in which desistance was rewarded with support to find employment, education, and healthcare, including treatment for substance abuse.

More recently, and despite the fall in police-recorded convictions for violence, Glasgow has experienced a dramatic increase and now has the highest levels of drug-related deaths in Europe due to opioid misuse. Young men in areas like GE are increasingly involved in the underground drug economy since our survey. The observation that progressive reduction of violence followed by a dramatic escalation in deaths from drug misuse suggests that the associations we originally observed between drugs and violence were largely indirect, likely to have had common causes, but may now have changed. Violence may have become increasingly purposeful and instrumental in the distribution of drugs, whilst becoming less prevalent overall. Nevertheless, the forces previously driving these adverse outcomes of violence and drug misuse at the time of the survey are likely to be still in operation and will require multi-agency approaches to interventions in future.

## Supplementary Information

**Additional file 1.**

## Data Availability

The datasets generated and/or analysed during the current study are available from the corresponding author on reasonable request.
