# Supplementary material for: Interpersonal violence in a deprived Scottish urban area with aggregations of physical health risks and psychiatric morbidity: an ecological study
Source: BMC Public Health. 2021 Jun 12;21:1121. doi: 10.1186/s12889-021-11167-z (PMC8196543; doi:10.1186/s12889-021-11167-z)
Supplement: Supplementary file 1 — Additional file 1. [file 12889_2021_11167_MOESM1_ESM.docx]

**The First Men’s Modern Lifestyles Survey.**

The ‘Men’s Health and Modern Lifestyles Survey’ was carried out in 2009 by ICM for Queen Mary, University of London and was funded by The Maurice and Jacqueline Bennett Charitable Trust. The sample comprised 3025 men aged 18–64 years living in England, Wales and Scotland. A one-stage survey sought to interview a geodemographically representative sample of the male population of the United Kingdom through a random location methodological approach. Random location techniques utilize a full selection of geographic areas to be visited by interviewers, allied to quota sheets showing exactly who they must approach and interview within their target geography. This procedure necessitated the use of profiling statistics from the then most up-to-date Census (2001).

Within each Government Office Region, all output areas (OA) (averaging 150 households, and about which all demographic profiling information is known) were selected and listed in descending order of ACORN (A Classification Of Residential Neighbourhoods) (1) type to place the most affluent OAs at the top of the list and the least affluent at the bottom. This applies a purely random variable into the selection of sampling locations. The total number of eligible male adults in each OA were then cumulated down the list. Using a random start and fixed sampling interval, the required number of OAs were selected. This process produces a sample of OAs with a probability of selection proportionate to size and was designed to produce a representative sample by ACORN type. A total of 250 OAs were selected, with interviewers required to achieve 12 interviews with eligible targets

at each. All addresses that lay within selected OAs were potentially available for interview. With OA information cross-referenced against full address lists, interviewers were supplied with every single address that was eligible within each OA. A quota sheet was provided for each selected OA, which reflected the actual composition of eligible residents according to standard demographic criteria. These would include socio-demographic characteristics such as gender, ethnicity and working status (in addition to age). Interviewers were required to interview a sample profile that matched exactly that of the eligible OA population profile using the then up-to-date Office of National Statistics (ONS) population estimates information. This ensured that the sample was demographically representative at the micro-level, as well as geographically representative of males in the general population. If a participant refused to complete the questionnaire (approximately 23% of all participants approached), or was absent, another was located in the area with exactly the same demographic profile (age and social class) until the quota was filled.

The statistical reliability of this approach depended both on strictly defining the selection of the sampling points as well as in setting representative quotas at each point, and then meeting these quotas meticulously. Compliance with this procedure produced a fully representative data set. Self-report questionnaires were administered at home, with the respondent left to complete the questionnaire in their own time. The researcher either returned later that day or the next. Each questionnaire took approximately 45 minutes to complete. Participants were given £5 on completion of the questionnaire. A total of 3025 male adults completed the questionnaire. Study design and procedures were approved by the Queen Mary, University of London Human Research Ethics Committee.

**The Second Men’s Modern Lifestyles Survey.**

This study has been previously described briefly in a published paper (2) and was part of a research programme into risk assessment of violence and identification of risk factors funded by UK National Institute of Health Research (3). It was one component of the overall programme and was aimed to identify risk factors at the population level. In some cases, analyses were carried out combing data with national surveys of psychiatric morbidity that were available from the UK Department of Health (3).

The survey was carried out in 2011 based on random location sampling and using the same method as the First Men’s Modern Lifestyles Survey.. Individual sampling units (census areas of 150 households) were randomly selected within British regions in proportion to their population to derive a representative sample of young men (18–34 years) from England, Scotland and Wales. In this second survey, however ,there were four additional, boost surveys over-sampling young Black and minority ethnic men, and those from lower social grades. Two boost surveys oversampled from output areas in locations characterised by high gang membership, violence, and social exclusion: the London Borough of Hackney and Glasgow East, Scotland. The same sampling principles applied to each survey type. For the present study on the syndemic, we only included the representative sample from England, Scotland and Wales and the boost sample for Glasgow east.

The self-administered questionnaire piloted in the previous survey was adapted and informed consent obtained from respondents, as before. Participants were contacted in person by interviewers and, if agreeing to participate, completed the pencil and paper questionnaire in private and returned it to the interviewer. All participants were paid £5 for taking part in the survey and all questionnaires were anonymised.

Weights were constructed for each survey using Random Iterative Method (RIM) weighting to ensure representativity of the sample. All descriptive and subsequent statistical comparisons were based on weighted data.

(1) CACI Ltd. The ACORN user guide: The Consumer Classification.London: CACI Ltd; 2014.

(2) Coid J, Ullrich S, Keers R, Bebbington P, Destavola BL, Kallis C, Yang M, Reiss D, Jenkins R, Donnelly P (2013) Gang Membership, Violence and Psychiatric Morbidity. *American Journal of Psychiatry* **170**, 985-993.

(3) Coid JW, Ullrich S, Kallis C, Freestone M, Gonzalez R, Bui L, Igoumenou I, Constaninou A, Fenton N, Marsh W, Yang M, DeStavola B, Hu J, Shaw J, Doyle M, Archer-Power L, Davoren M, Osumili B, McCrone P, Barrett K, Hindle D, Bebbington P. Improving Risk Management for Violence in Mental Health Services: A Multi-Methods Approach. *NIHR Programme Grants for Applied Research (PGfAR*) No. 4.16. 2016. DOI: 10.3310/pgfar04160

***Quota Sampling and the 2011 Young Men's Health Survey - Report from ICM (Surveying Company).***

Individuals were recruited by proportional quota sampling. This is a standard method that entails setting quotas for participants on a range of demographic factors and ensures that the sample interviewed is representative of the population of interest. It is particularly useful when investigating hard-to-reach samples, sensitive subjects such as violence, sexual behaviour, etc, and where it is anticipated that certain sub-sections of the population are less likely to complete interviews or questionnaires (e.g. young, male, lower social class, ethnic minorities, in areas of socioeconomic deprivation).

Quota sampling offers an alternative to probability sampling and is often used in market research and national surveys and becomes necessary if there is no listing of all those eligible to be included. It is more efficient as recruitment and sampling can be focused in areas in which the desired population are resident but does require good census data on the characteristics by which the quota are set. This method is preferred if the costs of probability sampling would be prohibitive and where feasibility issues become prohibitive.

Information on number of questionnaires returned was not collected because it is not a requirement of the methodology used here. The 2011 Young Men's Health Survey – and previous waves of research – adopted an in-home ‘random location’ with respondent self-completion and interviewer pick-up. Random location is the most common form for social and public policy research surveys as it combines a rigorous methodology with relatively low costs.

In this particular survey, we used Output Areas as the principal sampling unit. OAs are the base unit of the Census outputs and are based on groups of postcodes that fit within the boundaries of electoral wards/divisions and parishes. OAs represent the lowest geographical level on which full information can be generated through Census output. Each OA contains approximately 150 households and interviewers were required to achieve a target of 13 interviews (i.e. questionnaire pick-ups) in each OA.

A quota sheet was provided for each selected grouped OA, which reflects the actual composition of OA residents according to standard demographic criteria. Interviewers were required to interview a sample profile that exactly matches that of the grouped OA population profile using Census population information. This ensures that the sample is demographically representative at the micro-level, as well as geographically representative.

Crucially, interviewers were not required to record the number of questionnaires handed out, meaning it is not possible to record a response rate. This is in contrast to a random probability (pre-selected) survey where interviewers are required to visit randomly selected households and interview randomly selected individuals. With this technique, the response rate is recorded and taken as a measure of quality and robustness of the sample.

However, it is possible to work out an indicative response rate to the 2011 Young Men's Health Survey. ICM interviewers are required to leave a minimum of three addresses between calls, as well as include evening and weekend visits so that they maximise the chance of meeting their quotas and to avoid clustering in a single street. As such, it is reasonable to assume that interviewers distributed questionnaires to no more than a quarter of the households in each OA. This being the case, an approximate response rate would be around 35% (i.e. 13 completed surveys per point from 37.5 households visited). Nonetheless, this is an approximation and cannot be regarded as a true response rate.

RIM (Random Iterative Method) weighting is a technique commonly used to weight market research data to known targets, eg age groups regions, gender, and specifically to each variable (question) independently. The technique allows the analyst to adjust multiple characteristics in the dataset all at the same time in a way that ultimately keeps the different characteristics proportionate as a whole.

RIM weighting is a special form of target weighting. It can be a practical tool to use when there are targets (or populations) to which we wish the data for two or more variables, but not targets for the interlocking cells for these two or more variables. These are known as ‘rim weighting targets’. There may be more than two variables, which is where rim weighting is likely to be the chosen method. RIM weighting works by what is known as an iterative target weighting process. In other words, the software will calculate targets for the first rim and, after applying this weighting factor, it is highly improbable that the precise target percentages for one variable (eg gender) would be achieved. As the programme performs the iterations, the data gets closer and closer to the targets.

ICM uses Quantum, one of the most widely used tabulation and data packages in the survey research industry.

Translation by interviewers was not allowed which meant that to complete questionnaires the participants had to be English speakers.
